# A novel nomogram for predicting subsyndromal delirium in critically ill patients with diabetes

**DOI:** 10.3389/fendo.2026.1916733

**Published:** 2026-09-14

**Authors:** Changyun Wei, Hui Wang, Jian Gao, Qin Zhu, Yushan Guan, Di Xiao, Hao Wang, Meng Su, Yunyi Le

**Affiliations:** 1 Department of Emergency, Peking Union Medical College Hospital, Chinese Academy of Medical Sciences & Peking Union Medical College, Beijing, China; 2 Department of Endocrinology and Metabolism, Peking University Third Hospital, Beijing, China

**Keywords:** critically ill, diabetes mellitus, nomogram, prediction model, subsyndromal delirium

## Abstract

**Background and aims:**

This study aimed to develop and validate a nomogram for predicting subsyndromal delirium (SSD) risk in critically ill patients with diabetes.

**Methods:**

Critically ill adult patients with diabetes were retrospectively included in the emergency department of Peking Union Medical College Hospital from January 2025 to December 2025. Univariate and multivariate logistic regression analyses identified independent predictors for SSD. A nomogram was constructed based on these predictors. The model’s performance was assessed by the area under the receiver operating characteristic curve (AUC), calibration curves, and decision curve analysis (DCA).

**Results:**

A total of 297 patients with diabetes were included and the overall incidence of SSD was 38.7%. Univariate and multivariate analysis identified six independent predictors: higher NRS score, RASS score, APACHE II score, PSQI score, random blood glucose level at admission, and lower Barthel index. The nomogram incorporating these factors demonstrated good discrimination, with an AUC of 0.922 (95% CI: 0.891-0.953). Calibration curves showed good agreement between predicted and observed probabilities, and DCA indicated favorable clinical utility.

**Conclusions:**

We developed and internally validated a nomogram for predicting SSD in critically ill patients with diabetes. The model may be useful for risk stratification, but prospective and external validation are required before clinical implementation.

## 1 Introduction

Delirium, an acute neuropsychiatric syndrome characterized by fluctuating disturbances in attention and cognition, is a common and serious complication in critically ill patients (1). In recent years, increasing attention has been paid to subsyndromal delirium (SSD), a preclinical or partial manifestation of delirium where patients exhibit one or more symptoms of delirium but do not meet the full diagnostic criteria (2). According to the Intensive Care Delirium Screening Checklist (ICDSC), a score of 1–3 points is defined as SSD, while a score ≥ 4 points meets the diagnostic criteria for full delirium.

More like accumulating evidence indicates that SSD, although generally considered less severe than delirium, is associated with clinically significant adverse outcomes. Patients with SSD experience significant short-term cognitive impairment, functional decline, and are at a substantially higher risk of progressing to delirium and mortality compared to those without delirium symptoms (3). Failure to recognize and manage SSD promptly may contribute to worsened outcomes, including prolonged mechanical ventilation, longer intensive care unit (ICU) stays, and increased healthcare expenditures (4).

Patients with diabetes mellitus (DM) constitute a large and growing proportion of the critically ill population in the emergency department (ED). They are particularly susceptible to acute brain dysfunction due to a confluence of risk factors. Hyperglycemia and hypoglycemia can directly impair cerebral metabolism and exacerbate neuroinflammation (5). Furthermore, patients with diabetes often have a high burden of comorbidities, such as hypertension and coronary artery disease, microvascular complications, and pre-existing cognitive impairment, all of which amplify their vulnerability to SSD (6, 7). Despite this heightened risk, there is a notable lack of tools specifically designed for the early prediction of SSD in this susceptible population. A DM specific model can more accurately stratify risk in this vulnerable population and guide diabetes-targeted interventions.

Current SSD prediction in clinical practice relies on identifying a multitude of risk factors, which can be broadly categorized into non-modifiable (e.g., advanced age, low education level) and modifiable factors (e.g., uncontrolled pain, sleep deprivation, use of sedatives, metabolic disturbances) (8, 9). While several assessment tools like the Confusion Assessment Method for the ICU (CAM-ICU) are validated for delirium screening, and they are diagnostic rather than predictive (10). A predictive model that integrates specific risk factors relevant to patients with diabetes could enable proactive interventions.

Therefore, the objective of this study was to develop and validate a nomogram based on readily available clinical parameters to predict the risk of SSD specifically in critically ill patients with diabetes. This tool aims to empower clinicians to identify high-risk individuals early, thereby enabling targeted prevention strategies to mitigate the onset and impact of SSD.

## 2 Methods

### 2.1 Study design and population

This retrospective study was conducted in the ED resuscitation room of Peking Union Medical College Hospital, a tertiary academic medical center in China. The study protocol was approved by the Institutional Review Board of Peking Union Medical College Hospital. we screened adult patients (age ≥ 18 years) admitted to the ED resuscitation room between January 2025 and December 2025. The inclusion criteria were: [1] confirmed diagnosis of diabetes mellitus (type 1 or 2) prior to admission; [2] length of stay in the ED resuscitation room > 24 hours. The exclusion criteria were: [1] pre-existing psychiatric disorders according to DSM-V criteria; [2] deep sedation (RASS score of -5 to -3) throughout the admission; [3] presence of delirium at admission or development of delirium; [4] evidence of organic brain injury on CT scan; [5] history of psychoactive drug addiction; [6] post-operative state from general anesthesia.

### 2.2 Data collection and assessments

All data were collected within 24 hours of admission (baseline assessment). SSD outcomes were assessed within 24 hours before discharge or transfer from the ED resuscitation room. A second researcher verified all data for accuracy on the day of discharge or transfer. The collected demographic and clinical characteristics data included​ age, sex, education level, marital status, payment source, comorbidities (hypertension and coronary heart disease), smoking history, drinking history, surgery history, transfusion history, fever at admission (body temperature >37.3 °C) and mechanical ventilation status. The ​assessment scales included: [1] SSD was assessed using the Intensive Care Delirium Screening Checklist (ICDSC), and was defined as an ICDSC score of 1–3 points (11); [2] pain was assessed using the Numerical Rating Scale (NRS) (12); [3] sedation level was assessed using the Richmond Agitation-Sedation Scale (RASS) (13); [4] disease severity was assessed using the Acute Physiology and Chronic Health Evaluation II (APACHE II) score (14); [5] activities of daily living was assessed using the Barthel index (15); [6] frailty was assessed using the FRAIL scale (16); [7] sleep quality was assessed using the Pittsburgh Sleep Quality Index (PSQI) (17). To ensure reliability, two charge nurses performed independent assessments simultaneously. If their scores agreed, the result was recorded. When two assessors disagreed, the deputy chief nurse performed a third independent assessment, and the third adjudicated score was used as the final result. Missing data were minimal in this cohort, accounting for less than 2% of all included variables. We applied a complete case analysis approach, which is methodologically appropriate given the very low proportion of missing values.

### 2.3 Development and internal validation of a nomogram

The nomogram was constructed using the rms package in R software (version 4.2.2) based on multivariate logistic regression model. The model’s discrimination was evaluated by the area under the receiver operating characteristic curve (AUC); Internal validation was performed using bootstrap resampling with 1000 repetitions on the final selected model. Variable selection was first performed on the full dataset to derive the final predictor model and bootstrap resampling was then applied to this fixed model to assess the stability of discrimination (AUC) and calibration. The bias-corrected AUC was derived from the bootstrap resamples. Decision curve analysis (DCA) was performed to evaluate the clinical net benefit of the nomogram across different threshold probabilities.

### 2.4 Statistical analysis

Statistical analyses were performed using SPSS 25.0. Continuous variables following a normal distribution were presented as mean ± standard deviation and compared using Student’s *t*-test. Non-normally distributed data were presented as median with interquartile range and compared using the Mann-Whitney *U* test. Categorical variables were expressed as frequencies and percentages and compared using the Chi-square test or Fisher’s exact test, as appropriate. Univariate logistic regression was first performed to identify variables associated with SSD. Variables with a *p*-value < 0.1 in the univariate analysis were entered into a multivariate logistic regression model using a backward stepwise selection method to identify independent predictors. Odds ratios (ORs) with 95% confidence intervals (CIs) were calculated. A two-tailed *p*-value < 0.05 was considered statistically significant.

## 3 Results

### 3.1 Patient characteristics and SSD incidence

A total of 297 critically ill patients with diabetes were included in the final analysis. The baseline characteristics of the total cohort, SSD group (n=115), and non-SSD group (n=182) are summarized in **Table 1**. The overall incidence of SSD was 38.7%. Compared to the non-SSD group, patients who developed SSD were significantly older (69.1 ± 14.6 *vs.* 66.0 ± 13.5 years, *p* = 0.001), more likely to be female (59.1% *vs.* 29.7%, *p* < 0.001), and had lower education levels (*p* = 0.001). Significant differences were also observed in smoking history, drinking history, fever at admission, and mechanical ventilation rate. Regarding assessment scores, the SSD group had significantly higher NRS, RASS, APACHE II, FRAIL and PSQI scores, and significantly lower Barthel index (all *p* < 0.001). Random blood glucose levels at admission were also significantly higher in the SSD group (14.2 ± 9.08 mmol/L *vs.* 11.2 ± 5.06 mmol/L, *p* < 0.001).

**Table 1:** Demographic data and clinical characteristics of the study population.

Characteristics	Total (n=297)	SSD (n=115)	Non-SSD (n=182)	*t/Z/χ2* values	*P* values
Age	67.0 ± 14.1	69.1 ± 14.6	66.0 ± 13.5	−3.486	0.001
Sex (n, %)				25.269	<0.001
male	175 (58.9)	47 (40.9)	128 (70.3)		
female	122 (41.1)	68 (59.1)	54 (29.7)		
Education level (n, %)				18.780	0.001
primary school and illiterate	65 (21.9)	40 (34.8)	25 (13.7)		
secondary school	153 (51.5)	52 (45.2)	101 (55.5)		
university education	79 (26.6)	23 (20.0)	56 (30.8)		
Marital status (n, %)				1.109	0.316
unmarried	9 (3.0)	5 (4.3)	4 (2.2)		
married	288 (97.0)	110 (95.7)	178 (97.8)		
Payment source (n, %)				1.923	0.166
self-pay	32 (10.8)	16 (13.9)	16 (8.8)		
medical insurance	265 (89.2)	99 (86.1)	166 (91.2)		
Hypertension (n, %)	202 (68.0)	82 (71.3)	120 (65.9)	0.934	0.334
Coronary heart disease (n, %)	97 (32.7)	32 (27.8)	65 (35.7)	1.994	0.158
Smoking history (n, %)	98 (33.0)	26 (22.6)	72 (39.6)	9.160	0.002
Drinking history (n, %)	77 (25.9)	15 (13.0)	62 (34.1)	16.217	<0.001
Surgery history (n, %)	33 (11.1)	7 (6.1)	26 (14.3)	4.796	0.029
Transfusion history (n, %)	24 (8.1)	10 (8.7)	14 (7.7)	0.096	0.757
Fever at admission (n, %)	63 (21.2)	35 (30.4)	28 (15.4)	9.551	0.002
Mechanical ventilation (n, %)	22 (7.4)	14 (12.2)	8 (4.4)	6.216	0.013
NRS score	1.0 (1.0, 2.0)	1.0 (1.0, 2.0)	1.0 (1.0, 2.0)	2.503	0.012
RASS score	0.0 (0.0, 1.0)	1.0 (0.0, 1.0)	0.0 (0.0, 0.0)	6.872	<0.001
APACHEII score	11.0 (7.0, 13.0)	13.0 (10.0, 15.0)	9.0 (6.0, 12.0)	6.251	<0.001
Barthel index	85.0 (65.0, 90.0)	65.0 (55.0, 75.0)	90.0 (85.0, 95.0)	−10.865	<0.001
FRAIL score	1.0 (1.0, 2.0)	2.0 (2.0, 2.0)	1.0 (1.0, 1.0)	9.669	<0.001
PSQI score	7.0 (5.0, 10.0)	9.0 (8.0, 11.0)	6.0 (4.0, 8.0)	8.711	<0.001
Random blood glucose	12.4 ± 7.06	14.2 ± 9.08	11.2 ± 5.06	−3.638	<0.001

Data are presented as mean ± SD, number (%) or median (interquartile range), as appropriate.

### 3.2 Univariate and multivariate logistic regression analyses​

The results of the univariate and multivariate logistic regression analyses are presented in **Table 2**. In univariate analysis, multiple factors were significantly associated with SSD. The multivariate analysis retained six independent predictors: NRS score (OR: 2.394, 95% CI: 1.203-4.764, *p* = 0.013), RASS score (OR: 3.110, 95% CI: 1.592-6.074, *p* = 0.001), APACHE II score (OR: 1.139, 95% CI: 1.024-1.266, *p* = 0.016), Barthel index (OR: 0.946, 95% CI: 0.920-0.973, *p* < 0.001), PSQI score (OR: 1.380, 95% CI: 1.189-1.602, *p* < 0.001) and random blood glucose (OR: 1.105, 95% CI: 1.042-1.171, p=0.001). Sample size was adequate for model development: 115 SSD events across 6 final predictors yielded approximately 19 events per predictor, satisfying the conventional 10-events-per-variable rule for logistic regression and supporting reasonable model stability.

**Table 2:** Univariate and multivariate logistic regression analyses for SSD risk factors.

Characteristics	Univariable OR (95% CI)	*P* value	Multivariable OR (95% CI)	*P* value
Age	1.031 (1.013, 1.050)	0.001		
Sex				
female	1 (reference)			
male	0.292 (0.179, 0.476)	<0.001		
Education level				
primary school and illiterate	1 (reference)			
secondary school	0.322 (0.176, 0.587)	<0.001		
university education	0.257 (0.128, 0.515)	<0.001		
Marital status				
unmarried	1 (reference)			
married	0.494 (0.130, 1.818)	0.301		
Payment source				
self-pay	1 (reference)			
medical insurance	0.596 (0.286, 1.245)	0.169		
Hypertension	1.284 (0.773, 2.132)	0.334		
Coronary heart disease	0.694 (0.417, 1.154)	0.159		
Smoking history	0.445 (0.263, 0.757)	0.003		
Drinking history	0.290 (0.156, 0.541)	<0.001		
Surgery history	0.389 (0.163, 0.928)	0.033		
Transfusion history	1.143 (0.490, 2.667)	0.757		
Fever at admission	2.406 (1.367, 4.236)	0.002		
Mechanical ventilation	3.015 (1.223, 7.435)	0.017		
NRS score	1.752 (1.178, 2.607)	0.006	2.394 (1.203, 4.764)	0.013
RASS score	4.033 (2.479, 6.560)	<0.001	3.110 (1.592, 6.074)	0.001
APACHEII score	1.206 (1.131, 1.285)	<0.001	1.139 (1.024, 1.266)	0.016
Barthel index	0.912 (0.894, 0.932)	<0.001	0.946 (0.920, 0.973)	<0.001
FRAIL score	11.977 (6.873, 20.870)	<0.001		
PSQI score	1.511 (1.356, 1.683)	<0.001	1.380 (1.189, 1.602)	<0.001
Random blood glucose	1.067 (1.027, 1.108)	0.001	1.105 (1.042, 1.171)	0.001

### 3.3 Development of the nomogram

The nomogram (**Figure 1**) was constructed using the six independent predictors identified in the multivariate analysis: NRS score, RASS score, APACHE II score, Barthel index, PSQI score, and random blood glucose at admission. For each patient, a point value is assigned for each predictor by projecting its value upwards to the ‘Points’ axis. The sum of these points yields a total score, which is then projected downwards to the ‘Risk of SSD’ axis to obtain the individual predicted probability of developing SSD.

**Figure 1:**
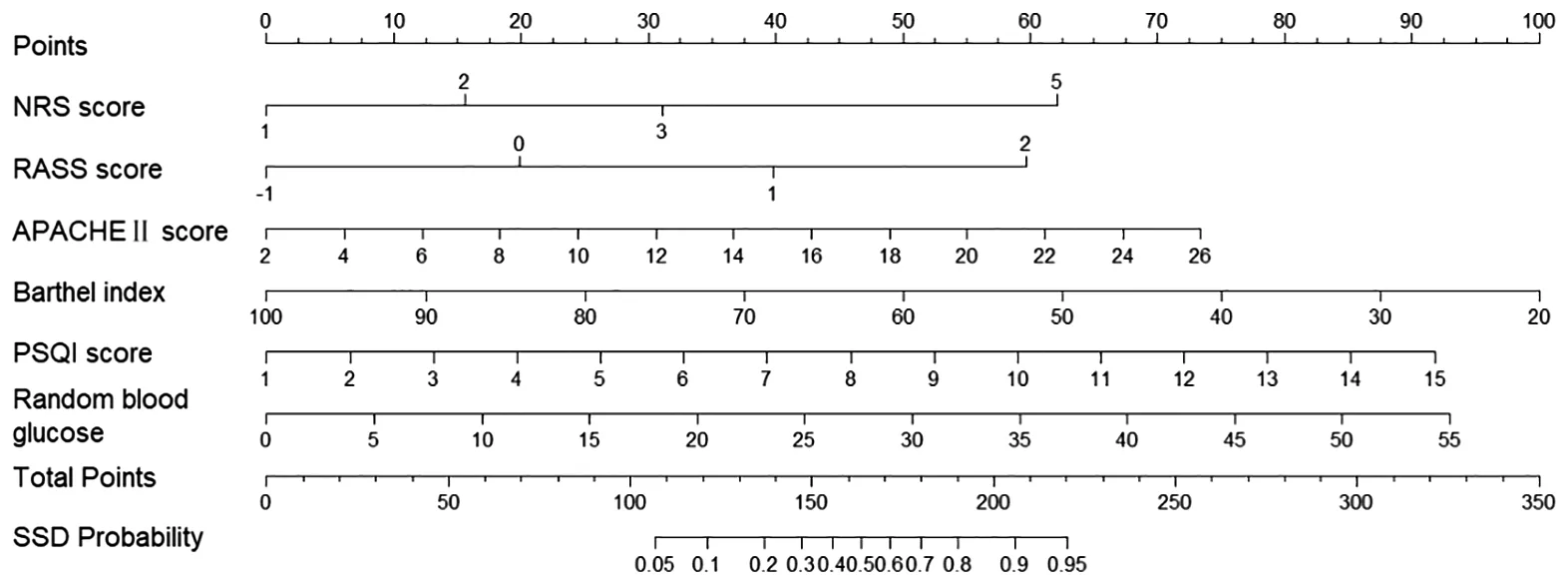
Nomogram to predict the probability of SSD in critically ill patients.

### 3.4 Internal validation of the nomogram

The nomogram demonstrated excellent discriminatory power. The model maintained good performance with the apparent AUC of 0.922 (95% CI: 0.891-0.953) (**Figure 2A**); after bootstrap internal validation (1000 resamples), the bias-corrected AUC was 0.908 (optimism = 0.014). The calibration curve for the probability of SSD showed good agreement between the nomogram’s predictions and actual observations (**Figure 2B**). Decision curve analysis revealed that using the nomogram to predict SSD risk provided a net benefit across a wide range of threshold probabilities (approximately 10% to 90%), confirming its potential clinical utility (**Figure 2C**).

**Figure 2:**
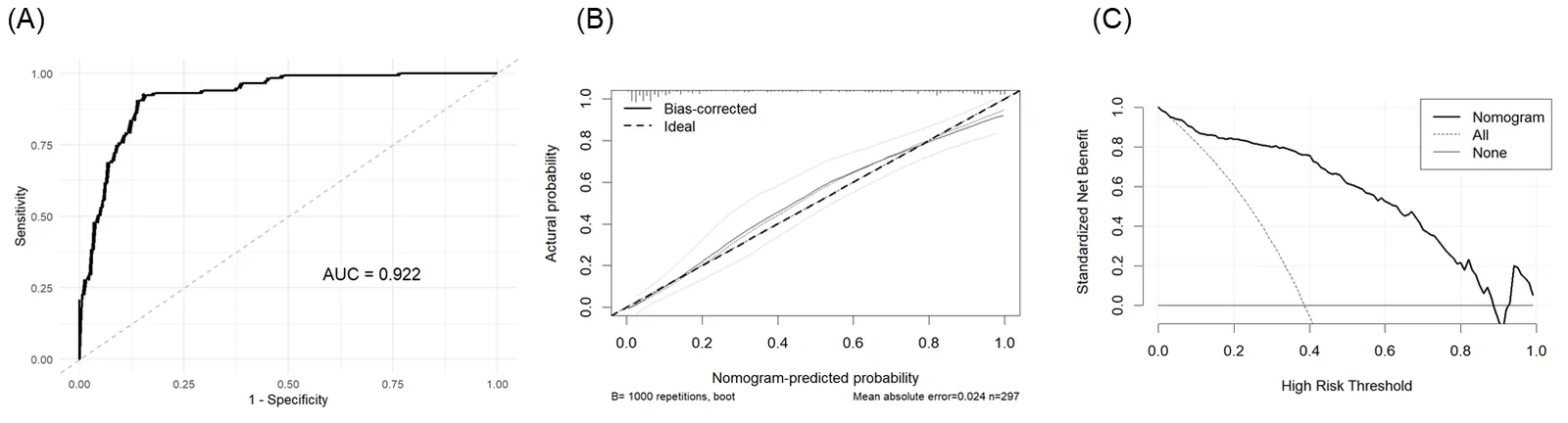
Internal validation of the nomogram: **(A)** ROC curves; **(B)** calibration plots; **(C)** decision curves.

## 4 Discussion

This study developed and internally validated via bootstrap resampling a nomogram specifically designed to predict the risk of SSD in critically ill patients with diabetes in the ED. The model incorporates six readily obtainable clinical variables—NRS score, RASS score, APACHE II score, Barthel index, PSQI score and random blood glucose—and demonstrated robust performance with good discrimination and calibration. Our findings underscore the multifactorial nature of SSD pathogenesis and highlight risk factors that are particularly prominent in the population. The core clinical value of this nomogram lies in its ability to identify high-risk patients early, and to guide targeted intervention on the four modifiable risk factors (pain, sedation, sleep, and blood glucose) to reduce the incidence of SSD.

The incidence of SSD in our cohort of patients with diabetes (38.7%) exceeded the previously reported range (8.97% to 34.5%) for general critically ill populations (18–20), supporting the premise that diabetes constitutes a significant vulnerability factor. This elevated risk likely stems from a confluence of factors, including chronic microvascular damage, baseline cognitive vulnerability, and a heightened inflammatory and stress response to acute illness (21).

The predictors identified in our final model are largely consistent with the established literature on delirium and SSD, yet their relative importance and specific implications in patients with diabetes warrant deeper analysis. The significant role of uncontrolled pain, as measured by the NRS score, is a pivotal finding. Pain induces a stress response characterized by cortisol and catecholamine release, which can lead to hyperglycemia and exacerbate neuroinflammation (22). In patients with diabetes with potential pre-existing neuropathy, pain may be more prevalent and poorly recognized, creating a vicious cycle. While the association between pain and delirium is well-documented in mixed populations (19, 23, 24), our study quantifies its independent contribution specifically in patients with diabetes, emphasizing that effective, multi-modal pain control is not just a comfort measure but a potentially important neuroprotective strategy requiring prospective confirmation in this group.

Our finding that a higher RASS score was a strong independent predictor aligns with numerous studies linking dysregulated arousal to delirium pathophysiology (25, 26). Agitation can be a manifestation of underlying SSD or a prodrome to delirium, while excessive sedation can predispose to brain dysfunction by reducing cholinergic tone. This reinforces the critical importance of targeting a “quiet awake” state as a cornerstone of delirium prevention bundles (27). Our results strongly suggest that this principle is equally critical in patients with diabetes.

The strong predictive value of pre-morbid functional status, measured by the Barthel index, echoes the well-established link between frailty and delirium vulnerability (28). Functional dependence is a marker of reduced physiological reserve, making it harder for patients to cope with the metabolic and inflammatory stresses of acute illness (29). It is noteworthy that the FRAIL score, while highly significant in univariate analysis, was not retained in the multivariate model. This suggests that the Barthel index, which captures actual performance of daily activities, may be a more robust indicator of physiological reserve relevant to SSD risk in this acute setting than a pure frailty screening tool. The correlation between these scales implies that the “frailty” phenotype significantly contributes to SSD risk through the pathway of functional impairment.

Poor sleep quality, captured by the PSQI score, was another significant predictor. Sleep deprivation disrupts the glymphatic system—the brain’s waste-clearance pathway—leading to the accumulation of neurotoxic metabolites like beta-amyloid, and promotes neuroinflammation (30). For patients with diabetes, who may already have altered sleep architecture due to autonomic neuropathy or comorbid conditions, this additional insult could be particularly detrimental. Our results add to the growing body of evidence supporting the implementation of non-pharmacological sleep promotion protocols (e.g., noise and light reduction, clustered care) as a standard of care for delirium prevention (31).

A key novel contribution of our study is the identification of random blood glucose at admission as an independent predictor, even after adjusting for illness severity and other confounders. Acute hyperglycemia is a common feature of critical illness and can induce cerebral oxidative stress, endothelial dysfunction, and disrupt the blood-brain barrier, creating a neurotoxic milieu in patients with diabetes (5, 32). While previous studies have linked hyperglycemia to delirium in general ICU populations (33), its role as a specific, modifiable risk factor for SSD in patients with diabetes has been less clear. Our data suggest that the dysregulated metabolic state itself, beyond just the burden of chronic disease, may contribute to acute brain dysfunction via multiple pathophysiological pathways. This finding strengthens the argument for early and careful glycemic control in the ED as a potential modifiable intervention to prevent SSD, though the optimal target range requires further investigation to avoid the risks of hypoglycemia.

Several limitations must be acknowledged. First, this was a single-center study, which may affect the generalizability of our findings and the SSD incidence and model performance may not be directly extrapolated to lower-acuity settings. External validation in diverse settings and patient populations is necessary. Second, while the sample size was adequate, a larger cohort would allow for more complex analyses and potentially identify additional subtle risk factors. Third, we used random blood glucose rather than HbA1c, which reflects long-term glycemic control. This means the model only reflects the impact of acute glycemic status on SSD risk. Future studies could investigate whether incorporating HbA1c improves prediction accuracy. Fourth, our study did not assess longer-term outcomes such as mortality and future studies could evaluate the nomogram’s ability to predict not only SSD but also subsequent clinical outcomes. Fifth, backward stepwise variable selection may produce slightly unstable coefficients and can exclude clinically relevant variables whose effects are mediated by stronger predictors; the final six-variable set should be viewed as an optimal predictive combination for this cohort, not an exhaustive list of all SSD risk factor. Sixth, the exclusion of patients who progressed to full delirium may have introduced selection bias by removing a clinically important high-risk subgroup. Consequently, our nomogram may not fully capture the risk profile of the broader critically ill diabetic population, and its performance in patients at the severe end of the delirium spectrum remains uncertain. Finally, the nomogram identifies risk but does not prescribe specific interventions. Prospective studies are needed to test whether a bundled intervention strategy targeting the modifiable factors in this model (e.g., pain, sedation, sleep, glucose) can effectively reduce SSD incidence in high-risk patients with diabetes identified by the nomogram.

## 5 Conclusions

In conclusion, we developed and internally validated a nomogram incorporating six readily available clinical variables (NRS score, RASS score, APACHE II score, Barthel index, PSQI score, and random blood glucose) to predict the risk of subsyndromal delirium in critically ill patients with diabetes. The model demonstrated good discrimination and calibration in this single-center retrospective cohort. This nomogram may serve as a potential tool for early risk stratification of SSD, helping clinicians identify high-risk patients. However, this study demonstrates only an association and does not establish that use of the nomogram leads to timely interventions, reduces SSD incidence, mitigates complications, or improves patient outcomes. Prospective multicenter external validation and interventional studies are required before the nomogram can be recommended for routine clinical implementation.

## Data Availability

The raw data supporting the conclusions of this article will be made available by the authors, without undue reservation.
