# Supplementary material for: A novel nomogram for predicting subsyndromal delirium in critically ill patients with diabetes
Source: Front Endocrinol (Lausanne). 2026 Sep 14;17:1916733. doi: 10.3389/fendo.2026.1916733 (PMC13616771; doi:10.3389/fendo.2026.1916733)
Supplement: Supplementary file 1 [file Supplementaryfile1.docx]

Supplementary Figure 1. Patient flow diagram

| Characteristics | B | S.E | Wals | 95% CI | *P* value |
| --- | --- | --- | --- | --- | --- |
| NRS score | 0.913 | 0.336 | 7.398 | 2.394 (1.203, 4.764) | 0.013 |
| RASS score | 1.134 | 0.323 | 12.324 | 3.110 (1.592, 6.074) | 0.001 |
| APACHEⅡ score | 0135 | 0.050 | 7.174 | 1.139 (1.024, 1.266) | 0.016 |
| Barthel index | −0.052 | 0.014 | 14.807 | 0.946 (0.920, 0.973) | <0.001 |
| PSQI score | 0.339 | 0.073 | 21.733 | 1.380 (1.189, 1.602) | <0.001 |
| Random blood glucose | 0.090 | 0.028 | 10.143 | 1.105 (1.042, 1.171) | 0.001 |
| Logit | −2.752 | 1.512 | 3.314 | 0.064 | 0.069 |

Supplementary table 1. Multivariate logistic regression analyses for SSD risk factor
